# SMAD7 regulates the canonical Wnt signaling through TGF-β cascade crosstalk and SMAD7/β-CATENIN transcription factor complex formation during tooth regeneration

**DOI:** 10.1038/s41368-025-00393-5

**Published:** 2026-01-06

**Authors:** Qiuyu Chen, Zhi Liu, Bohuai Zhou, Cheng Liang, Yiping Chen, Weidong Tian, Tian Chen

**Affiliations:** 1 State Key Laboratory of Oral Diseases & National Center for Stomatology & National Clinical Research Center for Oral Diseases & West China Hospital of Stomatology, Sichuan University, Chengdu, China; 2 Department of Cell and Molecular Biology, Tulane University, New Orleans, LA USA; 3 Department of Orthodontics, West China Hospital of Stomatology, Sichuan University, Chengdu, China

**Keywords:** Nuclear receptors, Regeneration

## Abstract

Tooth morphogenesis is orchestrated by a complex interplay of signaling pathways and transcription factors that control cell proliferation, apoptosis, and differentiation, with the Wnt/β-catenin signaling pathway playing a pivotal role. However, the comprehensive regulatory mechanisms of Wnt/β-catenin signaling remain largely unclear. Smad7, a key antagonist of the TGF-β superfamily, is essential for maintaining tissue homeostasis and ensuring proper cellular function. Our previous study has demonstrated that *Smad7* knockout in mice leads to impaired proliferative property of tooth germ cells, resulting in small molars. Here, we identified SMAD7 expression in human dental papilla and dental pulp, colocalized with β-CATENIN and cell proliferation-related proteins. RNA sequencing analysis revealed a significant reduction in Wnt signaling activity in *Smad7*-deficient mouse tooth germs. Using lentivirus transfection, we established *SMAD7*-knockdown human dental papilla stem cells, which manifested remarkably blunt proliferation rate, along with diminished Wnt signaling activity. In vivo transplantation investigations further revealed the indispensable role of SMAD7 in dentin formation. Mechanistically, we revealed that β-CATENIN interacts with P-SMAD2/3 and SMAD7 through co-immunoprecipitation and yeast two-hybrid assays. Inhibition of TGF-β pathway or disruption of SMAD7/β-CATENIN transcription factor complex formation potently impacted Wnt/β-catenin activities, indicating both direct and indirect regulatory mechanisms. These findings highlight the critical role of SMAD7 in the proliferation and differentiation of human dental stem cells, which could contribute to dental tissue regeneration and engineering.

## Introduction

Tooth morphogenesis is a tightly regulated process involving multiple signaling pathways and transcription factors, including Wnt/β-catenin and TGF-β, both critical for cell behavior and tissue organization.^1,2^ Wnt/β-catenin signaling, active throughout all developmental stages, controls cell proliferation and differentiation. Key Wnt genes, such as Wnt3, Wnt5a, Wnt7b, Wnt10b, are expressed as early as embryonic day 11 (E11) in mice, promoting dental placode formation.^3,4^ This pathway remains crucial at later stages (bud, cap, and bell) for cell proliferation and dynamics.^5,6^ Similarly, the TGF-β pathway also plays a vital role, with concentration-dependent effects in tooth development.^7,8^ Despite substantial insights into these individual pathways, the extent to which Wnt and TGF-β signaling pathways coordinate their actions, particularly in human dental mesenchymal stem cells, remains insufficiently characterized.

Smad7, a well-known intracellular antagonist of the TGF-β superfamily, inhibits TGF-β signaling by blocking Smad2/3 activation and promoting TGF-β receptor I (TGF-βRI) degradation.^9,10^ This regulatory role has been implicated in the development of various tissues and organs.^11,12^ In tooth morphogenesis, it has been reported that Smad7 was highly expressed in the dental epithelium and mesenchyme, acting as a positive regulator of cell proliferation by inhibiting TGF-β signaling.^13^ Interestingly, emerging evidence also suggests that Smad7 may intersect with Wnt signaling. For instance, in vascular smooth muscle cells (VSMCs), miR-195-5p targets Smad7 to promote the Wnt/β-catenin pathway, thus enhancing the osteogenic differentiation of VSMCs.^14^ These observations point to Smad7 as a potential integrator of TGF-β and Wnt signals in dental tissues, although the precise regulatory mechanisms remain unclear, and a deeper understanding of how Smad7 orchestrates these pathways may provide valuable insights into the molecular regulation of dental mesenchymal stem cell-mediated regeneration.

Adding further complexity, studies in non-dental tissues have demonstrated that Smad7 can form transcriptional complexes with β-catenin in a context-dependent manner. In skin, Smad7 binds to β-catenin and facilitates its degradation, thereby restricting the hair follicle formation regions.^15^ Conversely, in skeletal muscle development, this interaction promotes β-catenin nuclear translocation and enhances myogenic differentiation.^16^ However, whether such mechanisms operate in dental mesenchyme, and if Smad7 directly regulates β-catenin activity in human dental stem cells, has not been explored.

In this study, we conducted an in-depth investigation into the role of SMAD7 in modulating cell proliferation and differentiation in human dental mesenchymal stem cells (hDPSCs), with a particular focus on the underlying molecular mechanisms. Our findings demonstrate that SMAD7 serves not only as a coordinator of crosstalk between the canonical Wnt and TGF-β pathways, but also as a direct interactor with β-CATENIN through the formation of a transcriptional complex. Given the established importance of these pathways in controlling stem cell proliferation and differentiation, our findings reveal a dual regulatory mechanism by which SMAD7 modulates these critical cellular processes in odontogenic contexts. This mechanistic insight extends our understanding of SMAD7 function beyond tooth morphogenesis and underscores its potential as a molecular target in strategies for tooth regeneration and dental tissue engineering.

## Results

### SMAD7 is required for pulp-dentin complex regeneration of hDPSCs

**Fig. 1:**
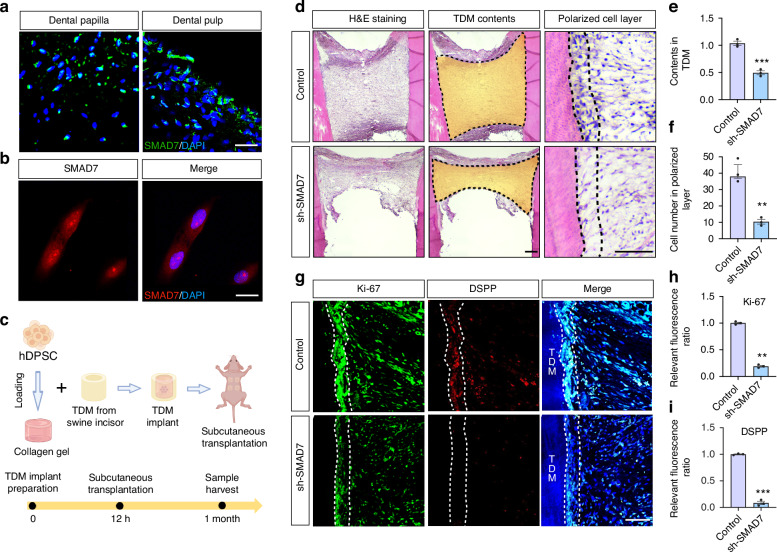
SMAD7 is widely expressed in human dental tissues and required for pulp-dentin complex regeneration of hDPSCs. **a** Immunofluorescent staining of SMAD7 in human dental papilla tissues and pulp tissues, respectively. Scale bars: 100 μm. **b** Immunofluorescent staining for SMAD7 in cultured hDPSCs. Scale bars: 30 μm. **c** Schematic of TDM subcutaneous transplantation in nude mice. **d** H&E staining of harvested TDMs of control and sh-*SMAD7* groups. The third column shows a magnified view of the polarized cell layer. Scale bars: 100 μm. **e** Quantification of the cell contents in (**d**). Statistical analysis was performed using Student’s *t* test (*n* = 3). ***P < 0.001. **f** Quantification of the polarized cell number. Statistical analysis was performed using Student’s *t* test (*n* = 3). ***P < 0.001. **g** Immunofluorescent staining for Ki-67 and DSPP in TDMs of control and sh-*SMAD7* groups. Scale bars: 100 μm. **h**, **i** Quantification of Ki-67- (**h**) or DSPP-positive (**i**) cells along the TDM shell in (**l**). Statistical analysis was performed using Student’s *t* test (*n* = 3). **P < 0.01, ***P < 0.001

Building on our previous findings that Smad7 acts as a key regulator in cell proliferation during murine tooth development,^13^ we sought to determine if SMAD7 is similarly expressed in human teeth. For this purpose, we harvested dental papilla tissues from developing third molars and dental pulp tissues from orthodontic subjects requiring tooth extraction, followed by immunofluorescent analysis (Fig. 1a). Indeed, SMAD7 was found strongly localized in the root furcation and apex of the dental pulp, with even higher levels in the dental papilla, implying that SMAD7 are involved in the regulation of development and maturation of human dental mesenchyme (Supplementary Fig. S1a, b).

To further investigate the potential role of SMAD7 in human dental pulp-dentin complex formation, we isolated and cultured hDPSCs in vitro, given their robust odontogenic capabilities and frequent use in dentin regeneration research.^17^ Immunofluorescent staining confirmed SMAD7 expression in hDPSCs (Fig. 1b). Accordingly, we conducted loss-of-function experiments using lentiviral short hairpin RNA (shRNA)-mediated knockdown targeting *SMAD7*. A substantial reduction in SMAD7 expression was seen by immunoblotting and immunofluorescent assays, validating the efficiency of the transduction system (Supplementary Fig. S1g–i).

We subsequently examined whether reduced *SMAD7* expression in hDPSCs impacts dentin formation. To this end, swine-treated dentin matrices (TDMs) were employed to encapsulate the control and *SMAD7*-deficient hDPSCs to mimic the dental root microenvironment and were then transplanted subcutaneously into nude mice, respectively (Fig. 1c). At 4 weeks post-transplantation, histological analysis with H&E staining revealed a remarkable reduction in cell content within the TDM in the *SMAD7*-knockdown group compared to controls (Fig. 1d, e). Although neither group manifested fully regenerated pulp-dentin complex-like structures or microvessel formation, a discernible layer of polarized cells lining along the TDM shell was observed only in the control hDPSCs, which closely resembled the odontoblasts along the pulp-dentin interface in native teeth (Fig. 1f). To further evaluate the tissue regeneration, dentin-specific marker DSPP and proliferation-related marker Ki-67 were assessed. Notably, the controls displayed much stronger expression of both proteins, indicating higher levels of cell proliferation rate and odontoblast differentiation (Fig. 1g–i). When considered together, these data suggest that SMAD7 plays an indispensable role in regulating the regenerative functions of hDPSCs, and its inhibition adversely affects dentin formation in the TDM model.

### SMAD7 loss leads to reduced Wnt/β-CATENIN signaling activity and cell proliferation rate

To better understand the global molecular impact of *Smad7* deletion on tooth development, we previously generated *Smad7* null mice (*Smad7*^–/–^) and performed bulk RNA-seq analysis on E15.5 molars from *Smad7*^–/–^ mice and littermate controls (*Smad7*^+/–^). Gene ontology (GO) analysis of the transcriptomic data revealed that genes downregulated in *Smad7*^–/–^ teeth were primarily associated with cell proliferation and cell cycle G1/S transition, whereas the upregulated genes were linked to the TGF-β signaling pathway (Supplementary Fig. S2a).^13^ Of note, the data also pointed to a significantly attenuated Wnt/β-catenin signaling activity, suggesting that Smad7 may mediate the canonical Wnt signaling during tooth development.

**Fig. 2:**
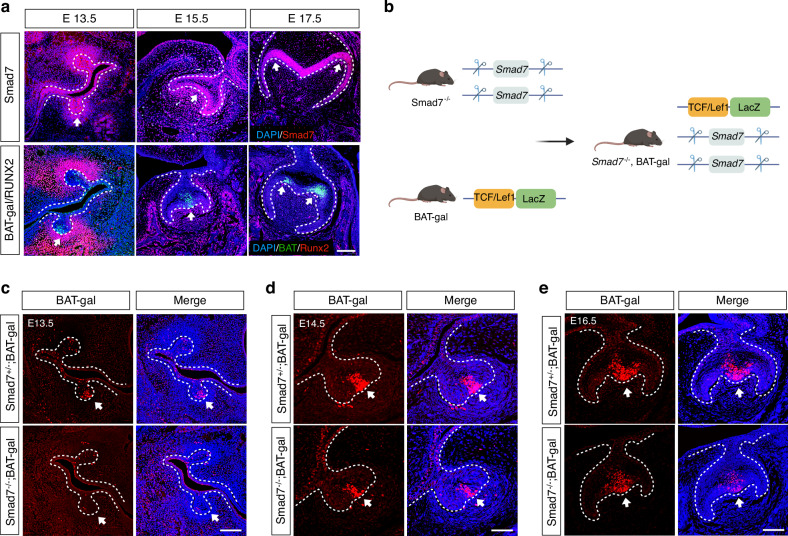
Smad7 is highly correlated with the Wnt/β-catenin signaling during mouse tooth germ development. **a** Immunofluorescent staining for Smad7, X-gal, and Runx2 in E13.5, E15.5, and E17.5 BAT-gal mouse molars. The white arrows pointed to the co-expression regions of Smad7 and BAT-gal. **b** Schematic of the construction of *Smad7*^−^^/^^−^;BAT-gal mice. **c**–**e** Immunofluorescent staining for X-gal in molars of *Smad7*^+/^^−^;BAT-gal, and *Smad7*^−^^/−^;BAT-gal mice at E13.5 (**c**), E14.5 (**d**), and E16.5 (**e**)

To determine the correlation between Smad7 and Wnt pathway, we utilized the BAT-gal reporter allele, which enables real-time tracking of Wnt/β-catenin signaling activity^18^ and conducted immunofluorescent staining on molar tooth germs at distinct developmental stages, including the bud stage (E13.5), the early bell stage (E15.5), and the late bell stage (E17.5). Throughout these periods, Smad7 expression consistently overlapped with the Wnt signaling reporter (Fig. 2a). To investigate if Smad7 plays a role in modulating Wnt/β-catenin signaling cascade, we created a *Smad7*^–/–^;BAT-gal mouse line upon crossing *Smad7*^–/–^ and BAT-gal mice (Fig. 2b). Such mice exhibited the significantly blunt activity of Wnt/β-catenin signaling in the dental epithelium, demonstrating that Smad7 serves as an upstream regulator of the canonical Wnt signaling pathway in murine tooth development (Fig. 2c–e).

**Fig. 3:**
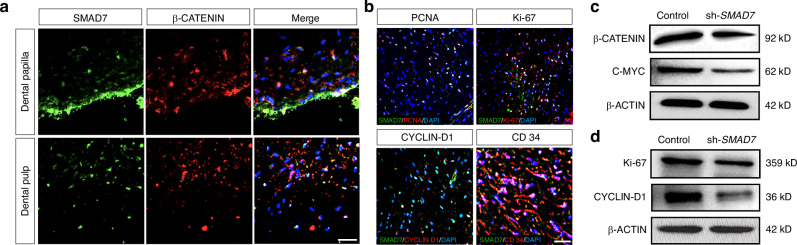
The expression of SMAD7 coincides with the Wnt signaling in human dental tissues. **a** Immunofluorescent staining for SMAD7 and β-CATENIN. **b** Immunofluorescent staining for SMAD7 and cell proliferation-related markers in human dental papilla, including PCNA, Ki-67, and CYCLIN D, along with the stem cell marker CD34. **c**, **d** Western blot analyses for C-MYC, β-CATENIN, CYCLIN-D1, and Ki-67 in control and sh-*SMAD7* hDPSCs. Statistical analysis was performed using Student’s *t* test (*n* = 3). *P < 0.05, **P < 0.01, ***P < 0.001. Scale bars: 100 μm

To ask whether SMAD7 is similarly related to Wnt signaling in human dental tissues, we performed immunofluorescent staining and found that SMAD7 was largely coincident with ACTIVE β-CATENIN in both dental papilla and pulp tissues (Fig. 3a). Interestingly, several crucial proliferation-related markers were also found co-localized with SMAD7, including CYCIN-D1, a known cell cycle regulator controlled by Wnt/β-CATENIN signaling (Fig. 3b). In parallel, Western blot analyses also demonstrated significantly reduced expression levels of Wnt signaling and proliferative markers in of the hDPSCs with SMAD7 knockdown compared to controls, such as C-MYC, β-CATENIN, Ki-67, and CYCLIN-D1 (Fig. 3c, d, Supplementary Fig. S2b–e). Taken together, these results indicate that Smad7 represents a positive regulator of both cell proliferation and the Wnt/β-catenin signaling pathway in developing teeth.

### *SMAD7* knockdown in hDPSCs results in reduced Wnt/β-catenin activity, impaired proliferation and migration, and increased apoptosis

**Fig. 4:**
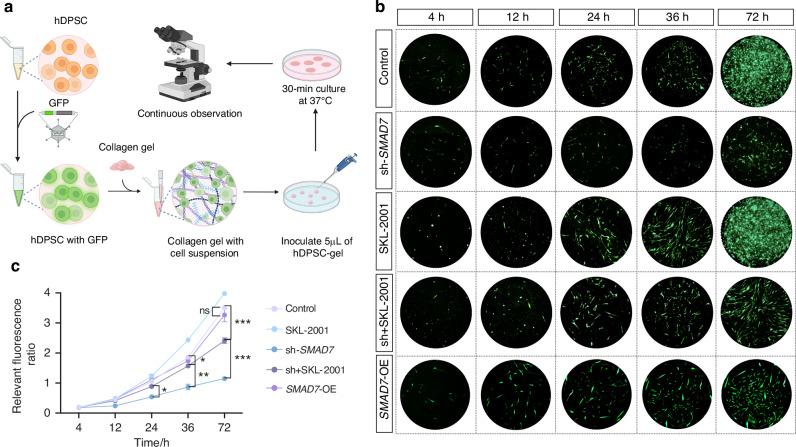
*SMAD7* knockdown significantly restrained the Wnt/β-catenin signaling pathway in hDPSCs, leading to compromised cell proliferation. **a** Illustration of the experiments in (**b**, **c**). **b** Cell sphere assays showing green fluorescence of hDPSCs at 4, 12, 24, 36, and 72 hours in the control, sh-*SMAD7*, SKL-2001, sh-*SMAD7* + SKL-2001 and *SMAD7*-OE groups. **c** Quantification of the relevant fluorescence ratio in (**b**). Statistical analysis was performed using two-way ANOVA, Tukey’s post hoc test (*n* = 3). *P < 0.05, **P < 0.01, ***P < 0.001

**Fig. 5:**
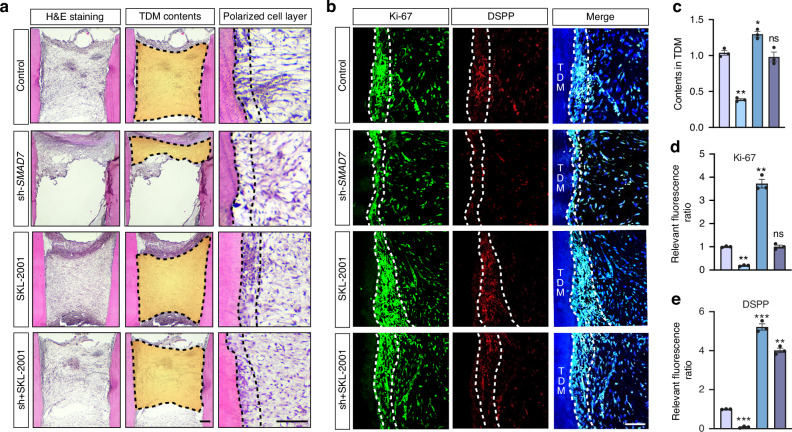
SMAD7 modulates the regenerative capacity of hDPSCs through Wnt/β-catenin pathway. **a** H&E staining of harvested TDMs from control, sh-*SMAD7*, SKL-2001, and sh-*SMAD7* + SKL-2001 groups. The third column shows a magnified view of the polarized cell layer. Scale bars: 100 μm. **b** Immunofluorescent staining for Ki-67 and DSPP in (**a**). **c** Quantification of the cell contents in (**a**). Statistical analysis was performed using one-way ANOVA, Dunnett’s post hoc test (*n* = 3). *P < 0.05, **P < 0.01, n.s., not significant. **d**, **e** Quantification of Ki-67- (**d**) or DSPP-positive (**e**) cells in (**b**). Statistical analysis was performed using one-way ANOVA, Dunnett’s post hoc test (*n* = 3). **P < 0.01, ***P < 0.001, n.s., not significant. The group designation of c, d, and e were indicated as that in Fig. 4c. Scale bars: 100 μm

Since the canonical Wnt signaling functions as a master regulator of cellular behavior, including proliferation, during tooth development in both mice and humans,^19^ the above data lead us to hypothesize that SMAD7 would be an important determinant of cell proliferation via modulating Wnt/β-catenin signaling. To examine the content to which SMAD7 loss alters cell proliferation in human dental cells, we evaluated the *SMAD7*-knockdown hDPSCs and found a profoundly decreased proliferation capacity with respect to SMAD7 deficiency, as evidenced by the substantially reduced cell number of hDPSCs labeled by GFP staining (Fig. 4a–c). However, when *SMAD7*-knockdown hDPSCs were treated with SKL-2001, a specific Wnt/β-CATENIN pathway agonist that stabilizes β-CATENIN by inhibiting its interaction with Axin,^20^ a significant recovery of cell proliferation was observed (Fig. 4b, c). In addition, hDPSCs transduced with *SMAD7*-overexpression vectors (*SMAD7*-OE) showed a modest increase in proliferative activity during the initial 24 hours; however, this effect was not maintained, and no significant difference was observed compared to the control group at subsequent time points (Fig. 4b, c). In parallel, flow cytometry assay showed a conspicuously lower percentage of *SMAD7*-knockdown hDPSCs in the S-phase of the cell cycle compared to controls, indicative of impaired cell division. Nevertheless, this deficit was restored by SKL-2001 administration (Supplementary Fig. S3a). Additionally, RT-qPCR analysis also manifested much lower expression levels of key Wnt signaling and cell proliferation markers, such as *C-MYC*, *CTNNB1* (encoding β-CATENIN), and *CCND1* (encoding CYCLIN-D1) in the absence of SMAD7, all of which became fully resumed following SKL-2001 administration (Supplementary Fig. S3b). To test if the increased Wnt signaling activity can also rescue the phenotype of SMAD7 loss in dentine formation in vivo, we subjected these plated cells to the transplantation experiments in nude mice analogous to the one used above. We found that SKL-2001 treatment potently rescued the cell content within TDM (Fig. 5a, c), accompanied by elevated Ki-67 and DSPP expression (Fig. 5b, d, e).

Apart from proliferation, *SMAD7*-knockdown hDPSCs displayed reduced migratory ability and increased apoptosis, further emphasizing the broad impact of SMAD7 on diverse cellular behaviors (Supplementary Fig. S3c–h). Altogether, these data support the importance of SMAD7/Wnt/β-CATENIN signaling axis not only in driving cell proliferation but also in orchestrating other fundamental cellular processes of hDPSCs.

### SMAD7 indirectly regulates Wnt pathway through inhibition of TGF-β signaling pathway

**Fig. 6:**
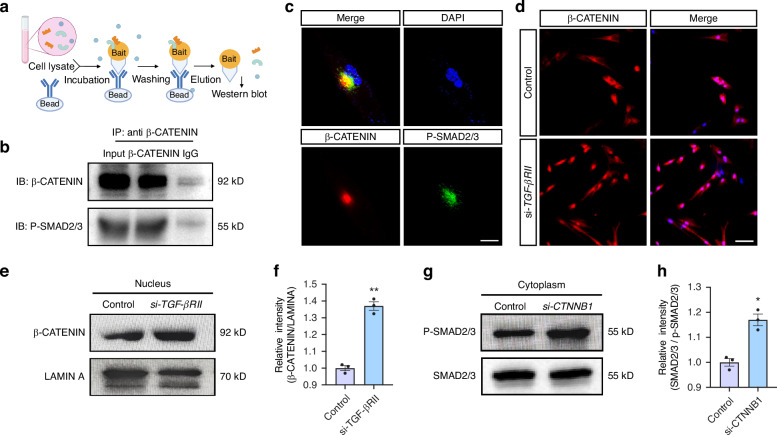
Crosstalk between the canonical TGF-β and Wnt signaling exists in hDPSC. **a** Schematic illustration of the Co-IP assay in (**b**). **b** Co-IP analysis demonstrating the interaction between β-CATENIN and P-SMAD2/3. **c** Immunofluorescent co-staining for β-CATENIN and P-SMAD2/3 in hDPSC. Scale bars: 30 μm. **d** Immunofluorescent staining for β-CATENIN in hDPSCs with or without si-*TGF-βRII* treatment. Scale bars: 50 μm. **e** Immunoblotting for nuclear β-CATENIN in hDPSCs with or without si-*TGF-βRII* treatment. **f** Quantification of the relative expression of β-CATENIN in (**e**). Statistical analysis was performed using Student’s *t* test (*n* = 3). **P < 0.01. **g** Immunoblotting for cytoplasmic P-SMAD2/3 in hDPSCs with or without si-*CTNNB1* treatment. **h** Quantification of the relative expression of P-SMAD2/3 in (**g**). Statistical analysis was performed using Student’s *t* test (*n* = 3). **P < 0.01

We next sought to determine the mechanism by which SMAD7 regulates the Wnt/β-CATENIN signaling cascade. Given that SMAD7 serves as a potent antagonist against TGF-β pathway, and a prior study has demonstrated that Smad7 improves the cell proliferation rate of murine tooth germ via the abolishment of the canonical TGF-β signaling,^13^ we started with examining the involvement of TGF-β signaling in the SMAD7/Wnt/β-CATENIN axis. To test the potential crosstalk between the two canonical signaling, we conducted a co-immunoprecipitation (Co-IP) assay and found a significant interaction between β-CATENIN and P-SMAD2/3 (Fig. 6a, b). Histologically, we observed a co-localization of phosphorylated SMAD2/3 (P-SMAD2/3), the hallmark of the canonical TGF-β signaling pathway, and active β-CATENIN in hDPSCs (Fig. 6c). As a consequence, we asked if a mutual regulation exists between Wnt and TGF-β signaling. We used the small interfering RNA (siRNA) to specifically block either TGF-β or Wnt signaling by targeting *TGF-βRII* (encoding TGF-β type II receptor) and *CTNNB1*, respectively (Supplementary Fig. S4a, b). Indeed, *TGF-βRII* knockdown resulted in enhanced expression level of nuclear β-CATENIN (Fig. 6d–f), whereas increased cytoplasmic P-SMAD2/3 expression was detected in the setting of *CTNNB1* inhibition (Fig. 6g, h), demonstrating an antagonistic relationship between the two pathways, wherein β-CATENIN prevents P-SMAD2/3 from translocating to the nucleus by binding with it in the cytoplasm.

**Fig. 7:**
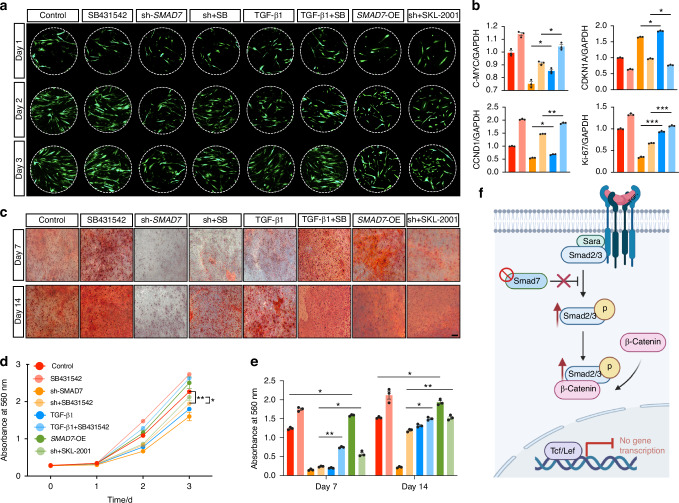
SMAD7 indirectly regulates Wnt pathway via suppression on TGF-β pathway. **a** Cell sphere assay showing green fluorescence of hDPSCs at Day 1, 2, and 3 in the control, SB431542, sh-*SMAD7*, sh-*SMAD7* + SB431542, TGF-β1, TGF-β1 + SB31542, *SMAD7*-OE, and sh-*SMAD7* + SKL-2001 groups. **b** RT-qPCR analysis of *C-MYC*, *CDKN1A*, *CYCLIN D1* and *Ki-67* expression in hDPSCs across treatment groups. Statistical analysis was performed using one-way ANOVA, Tukey’s post hoc test (*n* = 3). *P < 0.05, **P < 0.01. ***P < 0.001. **c** Alizarin red staining of hDPSCs in each treatment group. Scale bars: 50 μm. **d** Quantification of green fluorescence intensity in (**a**). Statistical analysis was performed using one-way ANOVA, Tukey’s post hoc test (*n* = 3). *P < 0.05, **P < 0.01. **e** Quantification of the Alizarin red density in (**c**). Statistical analysis was performed using one-way ANOVA, Tukey’s post hoc test (*n* = 3). *P < 0.05, **P < 0.01. ***P < 0.001. **f** Schematic of indirect regulation of SMAD7 on Wnt/β-catenin signaling pathway

To further clarify the involvement of SMAD7 in the crosstalk between TGF-β and Wnt signaling pathways, we examined the proliferative properties of *SMAD7*-deficient hDPSCs under conditions of modulated TGF-β signaling. By using recombinant TGF-β1 protein or the TGF-β receptor I inhibitor SB431542 (Supplementary Fig. S4c–f), we found that diminished cell proliferation (Fig. 7a, d) and Wnt/β-CATENIN signaling activity, as assessed by RT-qPCR assays (Fig. 7b). In contrast, SB431542 treatment significantly rescued these impairments (Fig. 7b, e). Notably, the Wnt agonist SKL-2001 exhibited an even greater restorative effect than SB431542, suggesting the central regulatory role of Wnt signaling in this context (Fig. 7b, e). Moreover, under osteogenic induction with a standard pro-differentiation cocktail, SB431542 administration markedly ameliorated mineralization in hDPSCs, particularly in *SMAD7*-knockdown and TGF-β1-treated cells (Fig. 7c, e). Interestingly, unlike the results observed in the proliferation assay, *SMAD7* overexpression significantly enhanced mineralization at both Day 7 and Day 14, with levels comparable to those seen in the SB431542 group (Fig. 7c, e). Nevertheless, neither pharmacological abolishment of TGF-β pathway nor TGF-β1 activation fully recapitulated the effects of SMAD7 loss, suggesting that SMAD7 may exert additional regulatory functions beyond TGF-β antagonism (Fig. 7c, e, Supplementary Fig. S4g, h). Collectively, these results support a model in which SMAD7 indirectly modulates Wnt/β-CATENIN signaling, at least in part, by inhibiting TGF-β pathway activity (Fig. 7f).

### SMAD7 also directly mediates Wnt pathway via the formation of a transcriptional complex with β-CATENIN

**Fig. 8:**
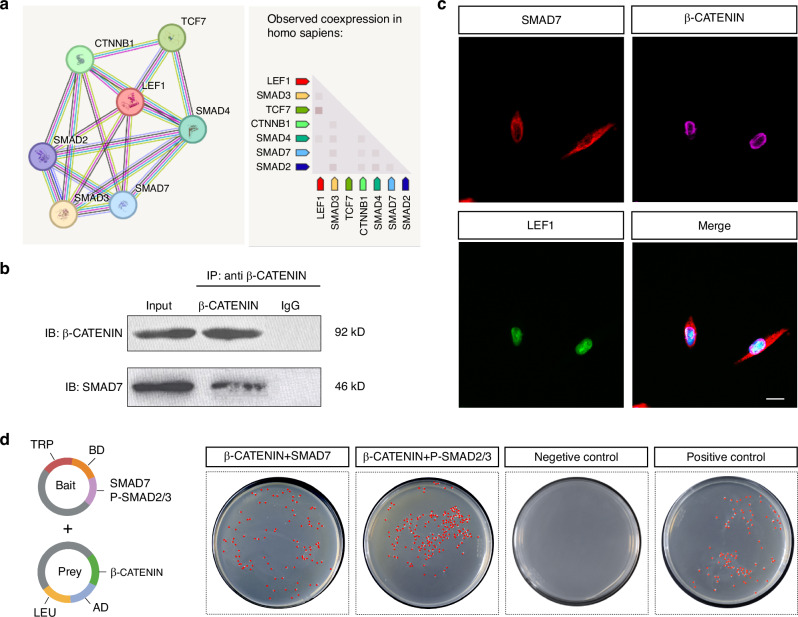
SMAD7 binds to β-CATENIN to form a transcriptional complex in hDPSC. **a** STRING database prediction showing potential interaction between SMAD7 and β-CATENIN. **b** Co-IP assay showing the interaction between SMAD7 and β-CATENIN. **c** Immunofluorescent co-staining for SMAD7, β-CATENIN, and LEF1 in hDPSCs. Scale bar: 30 μm. **d** Yeast two-hybrid assay demonstrating direct interactions among SMAD7, p-SMAD2/3, and β-CATENIN. Positive colonies are indicated by red spots

**Fig. 9:**
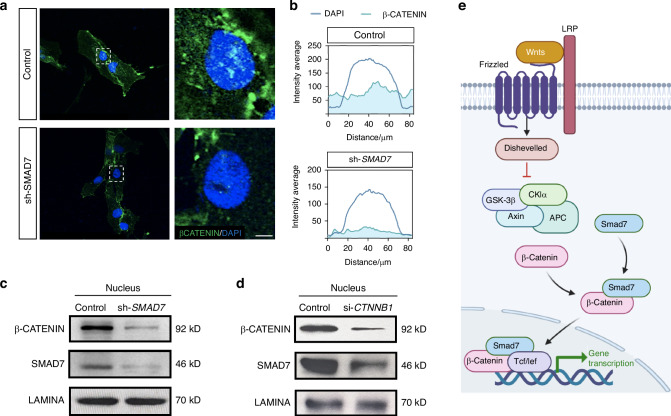
SMAD7 directly mediated the Wnt pathway by SMAD7/β-CATENIN transcriptional complex. **a** Immunofluorescent staining for SMAD7 and β-CATENIN in the nucleus of hDPSCs with or without sh-SMAD7 treatment. Scale bar: 15 μm. **b** Quantification of the fluorescence intensity of β-CATENIN in the nucleus. **c** Western blot analysis of nuclear β-CATENIN expression in control and sh-*SMAD7* hDPSCs. **d** Immunoblotting for nuclear SMAD7 expression in control and si-*CTNNB1* hDPSCs. **e** Schematic illustration of the direct regulatory mechanism by which SMAD7 modulates Wnt/β-catenin signaling

It has been reported that Smad7 can influence Wnt signaling via forming a transcriptional complex with β-catenin during the development of other organs/tissues.^15,16^ Thus, we further investigated if SMAD7 directly interacts with β-CATENIN in hDPSCs to promote its nuclear translocation and integrate with TCF/LEF. We firstly analyzed a protein database on STRING (https://string-db.org) and identified the potential co-expression and interaction between SMAD7 and β-CATENIN (Fig. 8a). Moreover, the spatial combination pattern of SMAD7 and β-CATENIN was predicted using IntAct (https://www.ebi.ac.uk/intact/) (Supplementary Fig. S5a). In support of this prediction, Co-IP assay demonstrated the formation of a SMAD7/β-CATENIN complex (Fig. 8b). In agreement with these observations, immunofluorescent staining of hDPSCs revealed a co-localization of SMAD7 and β-CATENIN (Fig. 8c). To further validate the results, we constructed recombinant plasmids (Supplementary Fig. S5b, c) and performed yeast two-hybrid experiments, which demonstrated the direct interactions between β-CATENIN and SMAD7, as well as between β-CATENIN and P-SMAD2/3 (Fig. 8d). Notably, knockdown of *SMAD7* resulted in a substantial reduction of nucleus-localized β-CATENIN, as revealed by immunofluorescent and Western blot analyses (Fig. 9a–c, Supplementary Fig. S5d, e). Similarly, knockdown of *CTNNB1* significantly impaired the nuclear translocation of SMAD7 (Fig. 9d, Supplementary Fig. S5f). Collectively, these data support the assertion that SMAD7 regulates Wnt/β-CATENIN signaling not only indirectly through the suppression of TGF-β signaling pathway but also directly by the formation of a transcriptional complex with β-CATENIN (Fig. 9e), which controls hDPSC proliferative property in the pulp-dentine formation and regeneration.

## Discussion

Cell proliferation is a fundamental biological process essential for tissue development and regeneration, including dental tissue engineering, which largely hinges on efficient and sustained cell proliferation to ensure proper tissue function and morphology.^21,22^ Our previous studies identified the Smad7/TGF-β/p21 axis as a critical modulator of epithelial proliferation during murine molar development. Loss of Smad7 in mice led to severely reduced cell proliferation and resulted in a smaller molar phenotype.^13^ Although *Smad7* null mice showed a remarkably compromised cell proliferation predominantly in dental epithelium, its role in dental mesenchyme cannot be neglected, for the reason that selective ablation of *Smad7* in the epithelium gave rise to significantly milder phenotype compared to the defects seen in *Smad7*^−^^/^^−^ tooth germs, indicating a broader functional role for Smad7 in the mesenchyme.^13,23^ Given the importance of dental mesenchymal stem cells in tooth regeneration, which are easily assessable, highly proliferative, and possess strong odontogenic differentiation capability, we thus investigated the expression and function of SMAD7 in hDPSCs in this study.^24,25^

Using a loss-of-function approach through shRNA-mediated knockdown, we found that SMAD7 deficiency resulted in significantly reduced hDPSCs proliferation and regenerative capability, indicating its essential role in pulp-dentin complex regeneration. Conversely, although *SMAD7* overexpression markedly enhanced the mineralization capacity of hDPSCs, it did not lead to a corresponding increase in proliferative activity. This discrepancy led us to propose that SMAD7 may function as a molecular facilitator of Wnt/β-catenin signaling, with its physiological expression already sufficient to sustain proliferative signaling homeostasis in hDPSCs. Under this model, reduction of SMAD7 disrupts this balance and impairs proliferation, whereas overexpression does not yield additional proliferative benefits once a functional threshold has been reached. This conclusion aligns with the concept of non-linear regulatory saturation commonly seen in regulatory networks during tissue development and stem cell modulation.^26,27^

Wnt/β-catenin signaling pathway is highly conserved across mammals and plays a central role in regulating cellular functions.^28,29^ Understanding the regulatory mechanisms governing this pathway is of great significance for unraveling the complex physiology underlying its activity. Here, we found that SMAD7 is more than merely an inhibitor of TGF-β pathway in promoting hDPSC proliferation, which is crucial in the regulation of odontogenic stem cell behaviors.^6,30^ Through transcriptomic profiling analysis of *Smad7*^−^^/^^−^ and control mice, we discovered that the activities of Wnt/β-catenin pathways were decreased in the absence of Smad7, suggesting that the canonical Wnt signaling pathway is intricately linked to Smad7 in controlling cellular processes within dental tissues. This result is intriguing because Smad7 was not previously related to Wnt signaling in dental tissues. Accordingly, we dissected the regulatory role of SMAD7 in modulating Wnt/β-catenin signaling both in transgenic mice in vivo and in human odontogenic stem cells cultured in vitro. Our findings demonstrate that SMAD7 positively mediates the Wnt/β-catenin signaling cascade, which contributes to enhancing cell proliferation. This conclusion is further supported by the results that SKL-2001 administration potently ameliorates the phenotype attributed to SMAD7 depletion.

Signaling pathways operate in concert to orchestrate various biological processes.^31,32^ Although key pathways such as Wnt, TGF-β, Bmp, Shh, and FGF have been extensively studied in tooth development, their intricate crosstalk remains inadequately explored, particularly the interplay between the Wnt and TGF-β pathways.^33,34^ Nevertheless, emerging evidence from other systems, such as skeletal muscle, has highlighted the existence of cross-regulation of these two pathways. The present study found that SMAD7 interacts with P-SMAD2/3 to impact Wnt/β-catenin signaling cascade by modulating TGF-β activity. Manipulation of TGF-β signaling cascade through ligand activation (TGF-β1), genetic suppression (siRNA targeting *TGF-βRII*), or pharmacological inhibition (SB431542) in the absence of SMAD7 markedly altered Wnt/β-catenin activity. Conversely, *CTNNB1* knockdown enhanced P-SMAD2/3 levels, revealing an antagonistic relationship between these pathways. These findings underscore the dual regulatory role of SMAD7, acting as a critical hub for the interactions between Wnt and TGF-β pathways in dental stem cells.

The significant contribution of transcription complexes to shape tissue development and regenerative processes has been well established.^35,36^ In addition to its regulatory interaction with P-SMAD2/3, we found that SMAD7 directly modulates nuclear β-CATENIN translocation. Interestingly, the function of SMAD7/β-CATENIN transcription complex appears to be tissue-dependent: while Smad7 binds to β-catenin to inhibit its function in the skin, Smad7 facilitates Wnt/β-catenin signaling during muscle differentiation. Unlike its inhibitory binding with P-SMAD2/3, we showed that SMAD7, in this case, promotes β-CATENIN nuclear translocation in hDPSCs, as mirrored by shRNA-mediated knockdown experiments. Additionally, loss of *CTNNB1* also led to a pronounced decrease in nuclear SMAD7, indicating a reciprocal interaction between these two factors. Given the integral roles of both Wnt/β-CATENIN and TGF-β signaling in vital physiological processes,^37,38^ it is conceivable that SMAD7 emerges as a critical mediator, coordinating the proliferation and differentiation of odontogenic stem cells.

Our current study establishes SMAD7/β-CATENIN as a key transcriptional regulator in hDPSCs. However, the specific Wnt target genes modulated by this complex, and the mechanisms by which these targets drive distinct cellular outcomes such as proliferation versus differentiation, remain unclear. It is plausible that SMAD7 influences β-CATENIN’s promoter-binding dynamics, potentially biasing downstream transcriptional responses toward lineage-specific programs. Future studies integrating ChIP-seq and epigenomic profiling will be critical to clarify these selective regulatory effects.^39^ Importantly, these mechanistic insights also inform the therapeutic modulation of SMAD7 in regenerative dentistry. For example, small molecules that stabilize the SMAD7/β-CATENIN complex or enhance its nuclear translocation could be designed to selectively activate Wnt-dependent differentiation pathways. Alternatively, CRISPR-based transcriptional regulation or miRNA delivery strategies might be used to fine-tune SMAD7 levels in specific contexts, improving control over hDPSC fate decisions. Collectively, understanding how SMAD7 shapes Wnt signaling at the transcriptional level lays a conceptual foundation for developing targeted interventions in dental tissue engineering.

In sum, the results presented here demonstrate a crucial role for SMAD7 in the regulation of cell proliferation in dental mesenchymal regeneration, functioning through both direct and indirect modulation of the Wnt/β-CATENIN signaling. Unmasking the intricate signaling dynamics is essential for advancing strategies in dental tissue engineering and improving regenerative therapies.

## Materials and methods

### Mice

Transgenic animal studies were conducted at Tulane University, and the construction of *Smad7*^−^^/^^−^ mice has been described previously.^13^ *Smad7*^−^^/^^−^;BAT-gal mice were obtained by mating *Smad7*^−^^/^^−^ mice with BAT-gal mice (JAX#005317, The Jackson Laboratory), the real-time reporter mice for Wnt signaling. The total number of transgenic mice used in this study was 24, with 6 in each group. The nude mice were purchased from Chengdu Dossy Experimental Animals.

All animal experiments and protocols adhered to the guidelines approved by the Institutional Animal Care and Use Committee (IACUC) of Tulane University and the Ethics Committee of the Experimental Animal Ethics Committee of West China Hospital of Stomatology, Sichuan University (WCHSIRB-D-2021-106).

### Acquirement of human dental papilla and pulp tissues

All participants provided informed consent after understanding the nature and purpose of this study, which was approved by the Ethics Committee of West China School of Stomatology, Sichuan University. Dental papilla tissues were obtained from six individuals, and pulp tissues were collected from five individuals, which were consisting of 12–25-year-old healthy men and women who underwent orthodontic treatments or received extraction surgery of developing third molar. Exclusion criteria included individuals with oral diseases such as dental caries, periodontitis, pulpitis, periapical periodontitis, or dental developmental anomalies.

### Tissue histology and immunofluorescence

Mouse samples (embryonic heads or newborn pups) were fixed overnight in 4% paraformaldehyde (PFA) at 4 °C, decalcified in 10% EDTA (pH 7.4) for 2–5 days, and dehydrated in 30% sucrose PBS at 4 °C. After embedding in OCT and freezing, the specimens were cryo-sectioned at 8 μm. Human dental papilla and pulp tissues from third molars were fixed in 4% PFA, dehydrated in graded ethanol solutions (70%–100%), infiltrated with paraffin, and embedded in paraffin blocks. The blocks were sectioned into 8 μm thick slices.

Histology and immunofluorescence staining were conducted as previously described.^40,41^ Detailed information for antibodies used in this study is provided in the Supplementary information.

### Cell culture

Dental papilla tissues were digested with 3 mg/mL type I collagenase (catalog no. C0130; Sigma-Aldrich, St. Louis) at 37 °C for 25 min to obtain single-cell suspensions. The cells were cultured in α-MEM supplemented with 2% FBS (catalog no. 0010, STEMCELL Technologies, USA), 1% mesenchymal growth supplement (catalog no. 7552, ScienCell, USA), and 1% penicillin/streptomycin (catalog no. 0503, STEMCELL Technologies, USA). DPCs P2 were used for all experiments. Cells were maintained at 37 °C with 5% CO_2_ in a humidified incubator (Thermo Fisher Scientific, USA), and the medium was refreshed every 3 days.

### RNA interference and pharmacological treatment of cells

The knockdown and overexpression of *SMAD7* in hDPSCs was achieved by introducing shRNA through infection with viral vectors. The shRNA plasmids were constructed, and HANBIO (Shanghai, China) performed lentivirus packaging. Optimum multiplicity of infection (MOI) and shRNA sequences were determined in a preliminary experiment. According to the results, sh-*SMAD7* 1 + 2 + 3 for knockdown and *SMAD7*-OE for overexpression with the MOI of 30 was selected in subsequent experiments (Supplementary Fig. S1c–i). The formal experiment was conducted as previously described.^42^ The knockdown of *CTNNB1* and *TGF-βRII* was accomplished using siRNA, also designed and constructed by HANBIO. The interference efficiency was evaluated by RT-qPCR and Western blot, and the information for primers and antibodies was provided in the Supplementary information.

### Pharmacological treatment of cells

SKL-2001, a specific Wnt/β-catenin pathway agonist, was used to restore the Wnt signaling in *SMAD7*-knockdown hDPSCs. For these experiments, hDPSCs from different groups were cultured in sixwell plates with 40 μmol/L SKL-2001 for 72 hours.

To inhibit the elevated TGF-β pathway, cells in different groups were treated with 20 μmol/L SB431542, a TGF-βRI competitive inhibitor, for 24 hours. Following treatment, western blot analysis was performed with the indicated antibodies to assess the effects on signaling pathways.

### Cell proliferation and migration assay

To assess hDPSC proliferation, we used a “cell sphere model”. Matrigel was diluted to 5 mg/mL and mixed with GFP-labeled hDPSCs (20 cells per μL). The mixture (5 μL) was seeded onto a plate and cultured at 37 °C for 30 minutes to solidify. Images of the cell spheres were captured at 4, 12, 24, 36, and 72 hours using a fluorescence microscope to monitor dynamic growth and proliferation.

### Cell migration assay

The wound healing assay was used to measure the cell migration abilities. In detail, plated hDPSCs in different groups were cultured in six-well plates until reaching confluence. A sterile pipette tip was then used to create a straight wound across the cell monolayer by removing cells from the designated area. The wells were gently rinsed with PBS to remove detached cells, and fresh culture medium was subsequently added. Images of the wound area were captured immediately after the scratch (time zero) and at 24- and 48-hour intervals during incubation, using a microscope. The healing process was quantified by measuring the scratch area at different time points, providing an assessment of cell migration.

### Flow cytometry

hDPSCs suspensions were transferred into tubes containing 70% ethanol for fixation and kept in fixative for more than 2 hours. The cells were then washed twice and resuspended in ice-cold PBS. Approximately 5 × 10^5^ cells were incubated with 1 mL PI/Triton X-100 staining solution containing RNase A for 15 minutes at 37 °C. Rat normal IgG (catalog no. 10700, Thermo Fisher Scientific, USA) was used as the negative control. After staining, the cells were washed three times with ice-cold PBS, resuspended thoroughly, and filtered through a 70-μm nylon mesh to remove clumps. The flow cytometer was set up for excitation with blue light and detection of PI emission at red wavelengths. Cell fluorescence was measured using a Becton-Dickinson Accuri C6 flow cytometer (BD Bio-sciences, USA). The pulse width-pulse area signal was used to distinguish between G_2_ cells and cell doublets, with the latter gated out. The data were analyzed using DNA content frequency histogram deconvolution software (FlowJo, version 7.6.1) to assess cell cycle distribution and related parameters.

### RNA preparation and RT-qPCR

Total RNA was extracted using the Trizol method (catalog no. 15596026, Thermo Fisher Scientific, USA). Following extraction, 1 μg of total RNA was used to synthesize first-strand complementary DNA (cDNA) with SuperScript II reverse transcriptase (catalog no. 18064014, Thermo Fisher Scientific, USA).

RT-qPCR was performed using SYBR Premix Ex Taq (catalog no. 172-5121, Bio-Rad, USA) to quantify gene expression. Transcript levels were examined by a 7500 Fast Real-Time PCR System. The primer sequences used in this study are provided in the Supplementary Table.

### Co-IP and western blotting

For Co-IP, lysates from 1 × 10^7^ hDPSCs were prepared and immunoprecipitated using IP buffer containing antibody-coupled agarose beads. The immunoprecipitated protein-protein complexes were then subjected to Western blot analysis. Labeled protein membranes were visualized and quantified using the Tanon 5200 system. IgG was used as a negative control for Co-IP experiments.

For western blotting, total protein lysates were prepared using lysis buffer (catalog no. KGP702-100, KeyGen Biotech, China) and quantified with the bicinchoninic acid Protein Assay Kit (catalog no. KGP902, KeyGen Biotech, China) according to the manufacturer’s instructions. Equal amounts of protein (20 μg per sample) were separated by SDS-polyacrylamide gel electrophoresis and transferred onto polyvinylidene difluoride membranes. The membranes were blocked with 5% skim milk in TBST (Tris-Buffered Saline and Tween 20) and subsequently incubated with primary antibodies. β-ACTIN was used as the internal control. After primary antibody incubation, membranes were treated with HRP-conjugated secondary antibodies and visualized using Clarify Western ECL Substrate (catalog no. 1705061, Bio-Rad, USA) according to the manufacturer’s protocol. Images were captured with ImageQuant LAS 4000 Mini (GE Healthcare Life Sciences), and protein band intensities were quantified by scanning densitometry using the ImageQuant TL software (GE Healthcare Life Sciences). Each experiment was performed independently, with at least three technical replicates for each sample, including treated and control groups. Details of the antibodies used in this study are provided below. For detailed information on antibodies, see the Supplementary information.

### Protein–protein Interaction (PPI) and spatial combination prediction

To visualize interactions between target proteins, the PPI prediction was conducted by typing the UniProt ID of SMAD7, β-CATENIN and P-SMAD2/3 on STRING (https://string-db.org/). After the interaction networks were generated, we chose the most credible network according to the credibility score and interaction type.

For the spatial combination prediction, IntAct (https://www.ebi.ac.uk/intact/) was used as the database framework. In detail, IntAct provided a Graph View to show the relationships between interacting proteins. Through visualization, the spatial binding domains could be clearly presented and analyzed. In the Graph View, through zooming, dragging, and adjusting the layout of models, the specific relationships between different interactions were captured.

### Yeast two-hybrid assays

The yeast two-hybrid screen was conducted twice using the Matchmaker^TM^ Gold yeast two-hybrid System (Clontech). The production of Y2H Gold yeast competent cells and recombinant plasmids was conducted as previously described.^43^

For validation, PGADT7-SMAD7 or PGADT7-P-SMAD2/3 prey plasmids were co-transformed with PGBKT7-β-CATENIN (bait vectors) into Y2H Gold competent yeast cells. Negative controls used pGBKT7-Lam and pGADT7-T, while positive controls used pGBKT7-53 and pGADT7-T. Positive results were identified as white colonies (marked as red), while negative results showed no growth.

### Subcutaneous transplantation experiments

To prepare TDM for cell implantation, incisor teeth from swine mandibles were extracted, and the periodontal ligament, outer cementum, inner pulp, and predentin were removed. The detailed preparation steps have been described previously.^40^ hDPSCs, sh-*SMAD7* hDPSCs, hDPSCs+SKL-2001, and sh-*SMAD7* hDPSCs+SKL-2001 were seeded into the prepared root canal pieces. To prevent cell leakage, the end of TDM near the skin was sealed with radiopaque calcium hydroxide composition (Dycal, catalog no. 10800, DENSPLY, USA). The TDM implants were then subcutaneously transplanted into immunocompromised nude mice and maintained for 4 weeks.

### Statistical analysis

Data are presented as the means ± S.E.M from at least three independent experiments. Statistical comparisons between two groups were performed using a two-tailed unpaired Student’s *t* test, while comparisons among three or more groups were analyzed using one-way ANOVA followed by Dunnett’s post hoc test (for comparisons relative to controls) or Tukey’s post hoc test (for multiple comparisons among groups). Statistical analyses were conducted using GraphPad Prism 10 (GraphPad Software Inc., San Diego, CA, USA). *P* < 0.05 was considered statistically significant for all tests.

## Supplementary information

Supplementary material

## Data Availability

All the data support the figures, and the other findings are available upon reasonable request to the corresponding authors.
