# Supplementary material for: SMAD7 regulates the canonical Wnt signaling through TGF-β cascade crosstalk and SMAD7/β-CATENIN transcription factor complex formation during tooth regeneration
Source: Int J Oral Sci. 2026 Jan 6;18:2. doi: 10.1038/s41368-025-00393-5 (PMC12770595; doi:10.1038/s41368-025-00393-5)
Supplement: Supplementary file 1 — Supplementary material [file 41368_2025_393_MOESM1_ESM.docx]

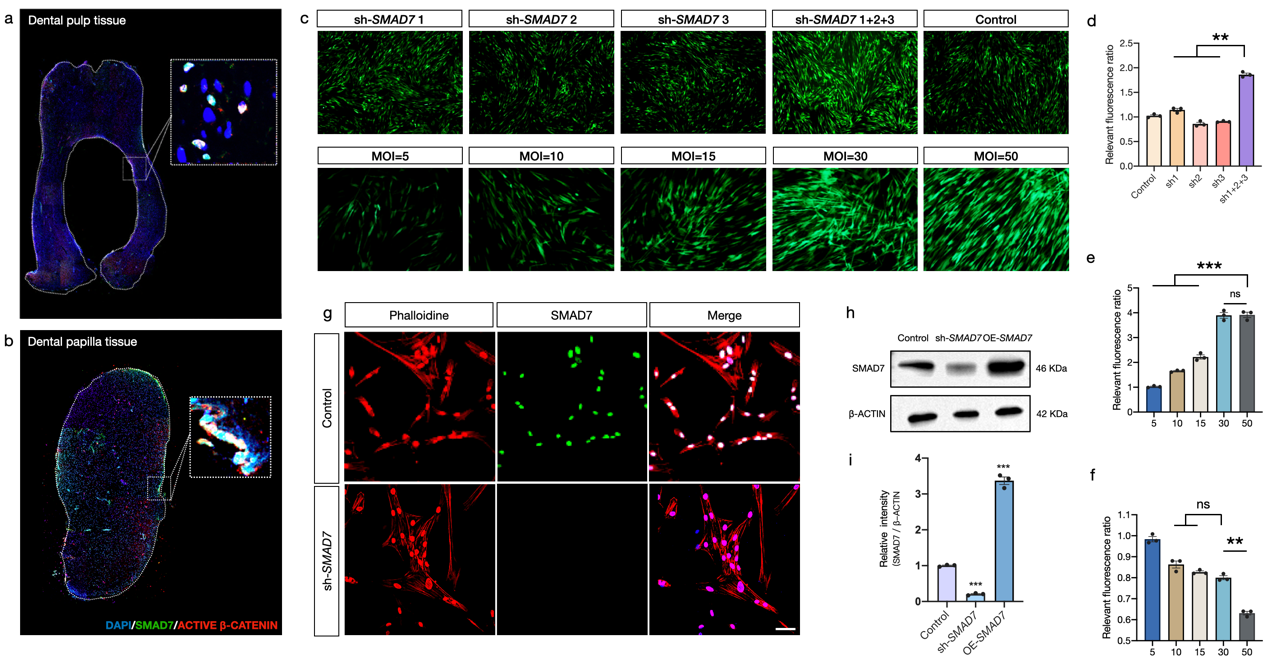


**Supplementary Fig S1.** Expression of SMAD7 in human dental tissues and establishment of *SMAD7* Knockdown/Overexpression hDPSCs. **a-b** Immunofluorescent staining of SMAD7 and β-CATENIN in human dental papilla and pulp tissues. The white boxes magnified the area where SMAD7 and β-CATENIN co-expressed. **c** Preliminary experiment of SMAD7-knockdown by shRNA under different RNA sequences and MOIs. **d** Relavant fluorescence ratio of transfected hDPSCs by different RNA sequences. Statistical analysis was performed using one-way ANOVA, Tukey’s post hoc test (*n* = 3). ***P* < 0.01. **e** Relavant fluorescence ratio of transfected hDPSCs under different MOIs. Statistical analysis was performed using one-way ANOVA, Tukey’s post hoc test (*n* = 3). ****P* < 0.001, n.s., not significant. **f** Cytotoxicity measurement, plotted from CCK8 assays, of shRNA at different MOIs. Statistical analysis was performed using one-way ANOVA, Tukey’s post hoc test (*n* = 3). ***P* < 0.01, n.s., not significant. **g** Immunofluorescent staining for SMAD7 in control and sh-SMAD7 hDPSCs. Scale bars: 100 μm. **h** Immunoblotting for SMAD7 in hDPSCs with or without OE-*SMAD7* treatment. **i** Quantification of the relative expression levels of SMAD7 in (**h**). Statistical analysis was performed using Student’s *t* test (*n* = 3). ****P* < 0.001.


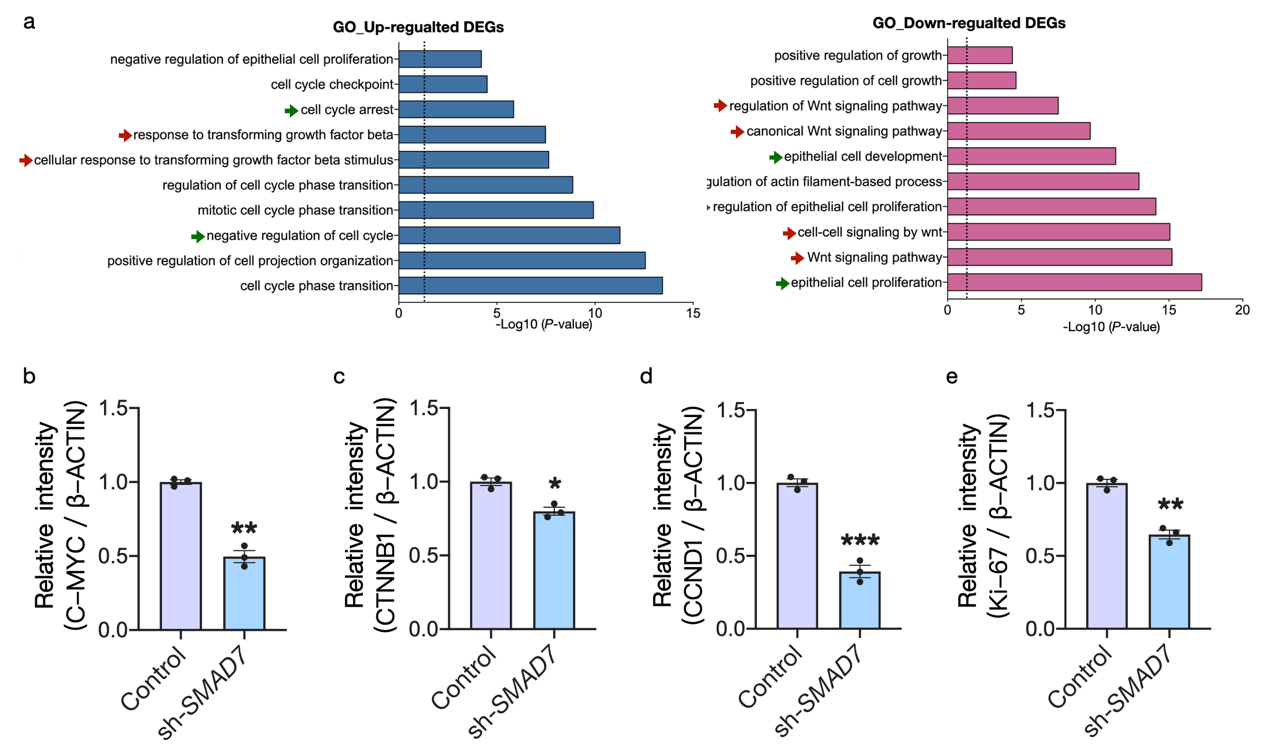


**Supplementary Fig S2.** SMAD7 is related to Wnt signaling in both mouse and human tissues. **a** GO analysis of upregulated (left panel) and downregulated (right panel) genes from RNA-seq datasets of E15.5 Smad7^–/–^ first mandibular molars of mice, as compared to controls. Green arrows indicate terms related to the cell cycle, and red arrows highlight terms associated with TGF-β and Wnt signaling pathways. **b-e** Quantification of the relative expression of C-MYC (**b**), β-CATENIN (**c**), CYCLIN-D1 (**d**), and Ki-67 (**e**) in (**Fig. 3c, d**). Statistical analysis was performed using Student’s t test (n = 3). **P* < 0.05, ***P* < 0.01, ****P* < 0.001.


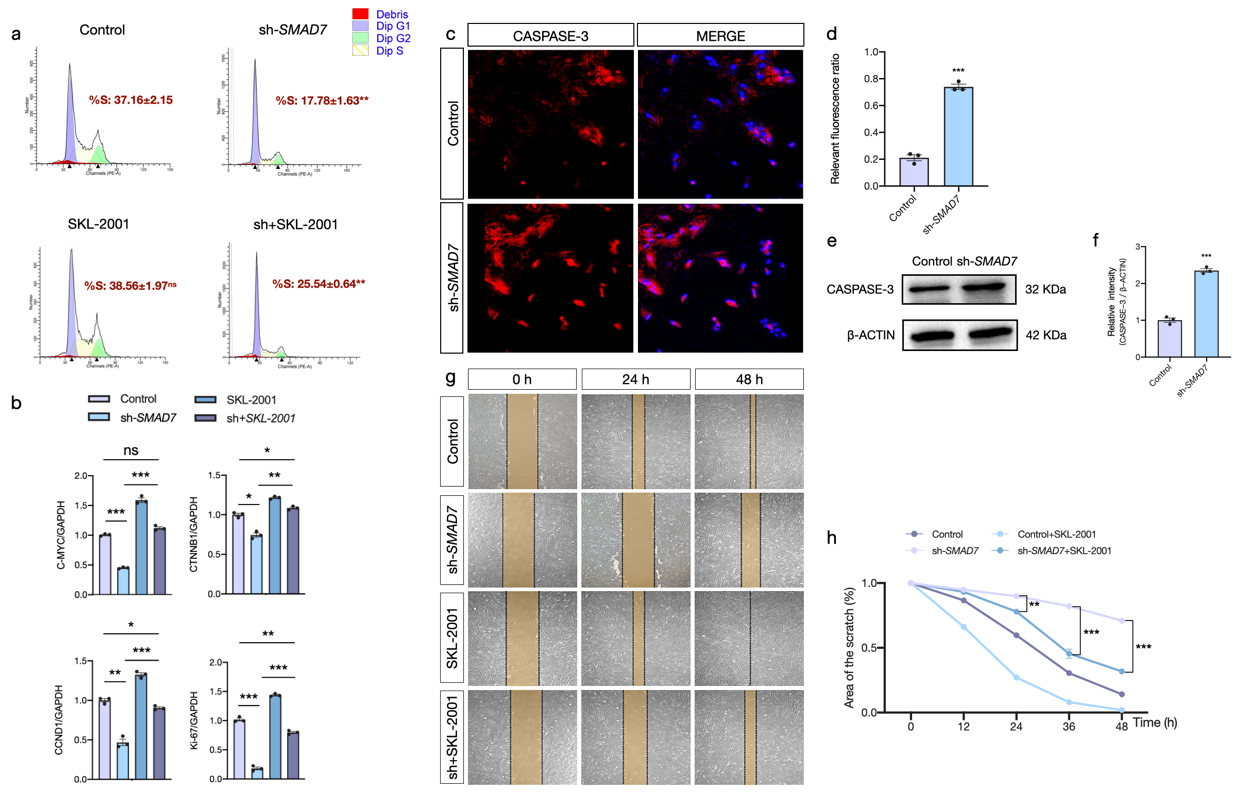


**Supplementary Fig S3.** Deficiency of *SMAD7* led to impaired cell cycle, cell migration and increased cell apoptosis via Wnt signaling. **a** Proportion of S Phase cells in different groups as assessed by the flow cytometry cell cycle assay. **b** RT-qPCR analysis of *C-MYC*, *CTNNB1*, *CYCLIN-D1*, and *Ki-67* expression in control and sh-*SMAD7* hDPSCs after 72h treatment with SKL-2001. Statistical analysis was performed using one-way ANOVA, Tukey’s post hoc test (*n* = 3). **P* < 0.05, ***P* < 0.01, ****P* < 0.001, n.s., not significant. **c** Immunofluorescent staining of CASPASE-3 in hDPSCs with or without sh-*SMAD7* treatment. **d** Quantification of CASPASE-3-positive cells. Statistical analysis was performed using Student’s *t* test (*n* = 3). ****P* < 0.001. **e** Immunoblotting for CASPASE-3 in control and sh-*SMAD7* hDPSCs. **f** Quantification of the relative expression of CASPASE-3 in (**e**). Statistical analysis was performed using Student’s *t* test (*n* = 3). ****P* < 0.001. **g** Wound healing assay of hDPSCs in control, sh-*SMAD7*, SKL-2001 and sh+SKL-2001 groups at 0, 24, 48 hours. **h** Quantification of the wound area in different groups. Statistical analysis was performed using one-way ANOVA, Tukey’s post hoc test (*n* = 3). ***P* < 0.01, ****P* < 0.001.


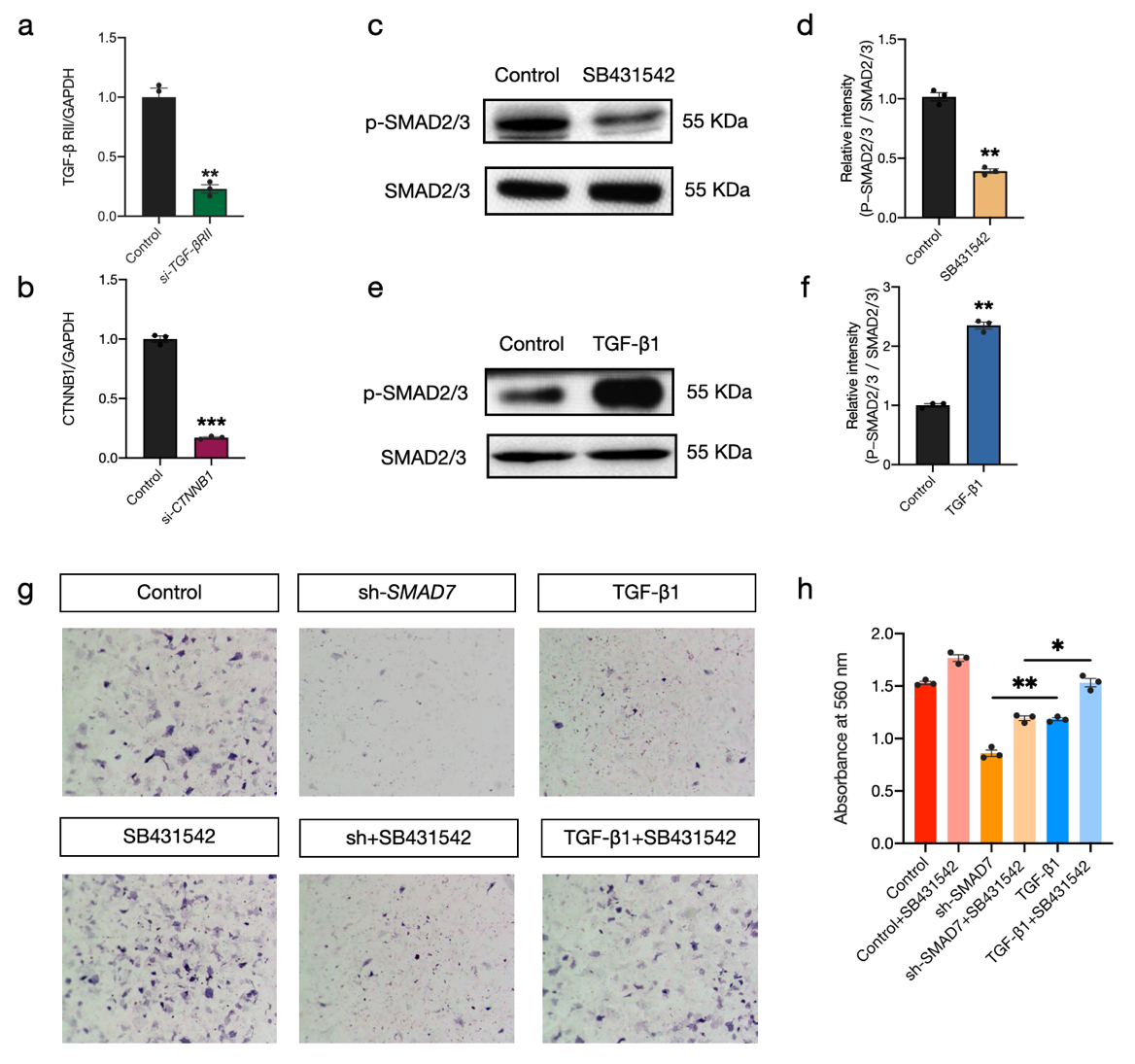


**Supplementary Fig S4.** TGF-β signaling regulation and SMAD7-mediated odontogenic differentiation in hDPSCs. **a** Quantitative reverse transcription polymerase chain reaction analyses of hDPSCs with or without si-*TGF-βRII* treatment. **b** Quantitative reverse transcription polymerase chain reaction analyses of hDPSCs with or without si-*CTNNB1* treatment. **c, e** Immunoblotting for P-SMAD2/3 in hDPSCs with or without SB431542 (**c**) /TGF-β1 (**e**) treatment. **d, f** Quantification of the relative expression of P-SMAD2/3 in immunoblotting. Statistical analysis was performed using Student’s *t* test (*n* = 3). ***P* < 0.01, ****P* < 0.001. **g** ALP staining of hDPSCs in *c*ontrol, SB431542, sh-*SMAD7,* sh+SB431542, TGF-β1 and TGF-β1+SB31542 groups. **h** Quantification of the ALP density in different groups. Statistical analysis was performed using one-way ANOVA, Tukey’s post hoc test (*n* = 3). **P* < 0.05, ***P* < 0.01.

**
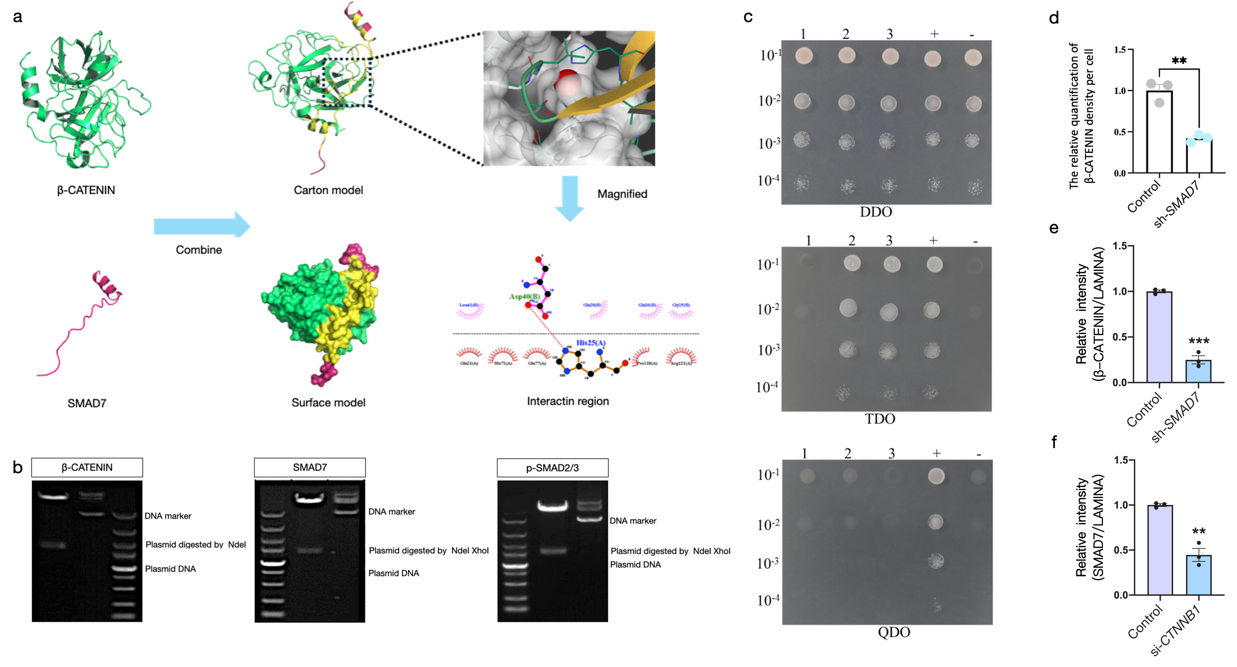
Supplementary Fig S5.** Prediction of the spatial binding site of SMAD7/β-CATENIN transcription factor complex and the construction of recombinant plasmids in yeast two-hybrid experiment. **a** Schematic of the direct combination pattern of SMAD7 and β-CATENIN predicted on IntAct. **b** Construction of recombinant plasmids used in the Yeast two-hybrid experiment. **c** Microbionation verification. 1: Y2H [PGBKT7-β-catenin + PGADT7]; 2: Y2H [PGBKT7-β-catenin + PGADT7-SMAD2]; 3: Y2H [PGBKT7-β-catenin + PGADT7-SMAD7]; +: Y2H [pGBKT7-53 + pGADT7-T]; -: Y2H [pGBKT7-lam + pGADT7-T]. **d** Quantification of β-CATENIN density per cell in (**Fig. 9a, b**). Statistical analysis was performed using Student’s t test (n = 3). **P < 0.01. **e** Quantification of nuclear β-CATENIN expression in (**Fig. 9c**). Statistical analysis was performed using Student’s t test (n = 3). ***P < 0.001. **f** Quantification of the relative expression of SMAD7 in (**Fig. 9d**). Statistical analysis was performed using Student’s t test (n = 3). ***P* < 0.01, ****P* < 0.001.

**Supplementary Table.** Primer sequences for RT-qPCR.

| Gene name | Forward primer (5’-3’) | Reverse primer (3’-5’) |
| --- | --- | --- |
| *SMAD7* | CCTGCCATTGTAGCGTCTTTC | CCCTTGGGAAGCCCATCT |
| *C-MYC* | CCCGCTTCTCTGAAAGGCTCTC | CTCTGCTGCTGCTGCTGGTAG |
| *CTNNB-1* | GAGGAGATGTACATTCAGCAGA | GTTGACCACCCCTGCATAG |
| *CCND1* | GCTGCGAAGTGGAAACCATC | CCTCCTTCTGCACACATTTGAA |
| *Ki-67* | TCCTTTGGTGGGCACCTAAGACCTG | TGATGGTTGAGGTCGTTCCTTGATG |
| *CDKN1A* | GGGAGCAGGCTGAAGGGT | CGGCGTTTGGAGTGGTAGAA |
| *TGF-βRII* | CTGTACATTGACTTCCGCAAG | TGTCCAGGCTCCAAATGTAG |
| *GAPDH* | CTTTGGTATCGTGGAAGGACTC | GTAGAGGCAGGGATGATGTTCT |

**Antibody Information**

Primary antibodies used in the study were:

1. Anti SMAD7 (Rabbit) 1:100 (IF), Sangon Bio, D160746
2. Anti SMAD7 (Mouse) 1:100 (IF), Santa Cruz, sc-365846

1:500 (WB), Santa Cruz, sc-365846

1-2 μg per 100 μg of total protein, Santa Cruz, sc-365846

1. Anti Ki-67 1:300 (IF), Abcam, ab15580

1:500 (WB), Abcam, ab15580

1. Anti DSPP 1:100 (IF), Santa Cruz, sc-73632
2. Anti β-CATENIN 1:100 (IF), Santa Cruz, sc-59737

1:500 (WB), Santa Cruz, sc-59737

2 μg per 100 μg of total protein, Santa Cruz, sc-5973

1. Anti PCNA 1:300 (IF), Abcam, ab29
2. Anti CYCIN-D1 1:100 (IF), Santa Cruz, sc-753

1:500 (WB), Santa Cruz, sc-753

1. Anti CD34 1:250 (IF), Santa Cruz, sc-7324

1:500 (WB), Santa Cruz, sc-7324

1. Anti P-SMAD2/3 1:50 (IF), Abcam, ab280888

1:1000 (WB), Abcam, ab280888

1:30 (IP), Abcam, ab280888

1. Anti LEF1 1:100 (IF), Abcam, ab85052

Secondary antibodies, all from Invitrogen and used at 1:500 dilution: 555 goat anti rabbit (A21428), 488 goat anti rabbit (A11008), 555 goat anti mouse (A21422), 444 goat anti mouse (A11001), and 555 Donkey anti Goat (A21432).
